# [4-(3-Amino-4-mehoxy-5-methylphenyl)-1-oxo-1*H*-phthalaz-2-yl] acetic acid hydrazide and its synergetic effect with KI as a novel inhibitor for low carbon steel corrosion in 0.5 M H_2_SO_4_

**DOI:** 10.1038/s41598-022-19057-z

**Published:** 2022-09-15

**Authors:** Rokaia Safwat Abdullah, Nehal A. Barghout, Sahar S. A. El-Sakka, Mohamed H. Soliman, Maher A. El-Hashash, Safaa Ragab, Ahmed El Nemr

**Affiliations:** 1 Environment Division, National Institute of Oceanography and Fisheries (NIOF), Kayet Bey, El-Anfoushy, Alexandria, Egypt; 2 Department of Chemistry, Faculty of Science, Suez University, Suez, Egypt; 3 Department of Chemistry, Faculty of Science, Ain Shams University, Cairo, Egypt

**Keywords:** Chemistry, Electrochemistry, Organic chemistry

## Abstract

We report the synthesis of novel [4-(3-amino-4-mehoxy-5-methyl phenyl)-1-oxo-1*H*-phthalaz-2-yl] acetic acid hydrazide (**APPH**), followed by its characterization using X-ray diffraction (XRD), Fourier transforms infrared (FT-IR) spectroscopy, ^1^H-NMR spectroscopy, and LC/MS. Further, the inhibition effect of the varying concentration of **APPH** on the corrosion of low steel (LCS) in 0.5 M H_2_SO_4_ was investigated by weight loss and electrochemical measurements at 30 °C. The percentage inhibition efficacy of APPH increased with concentration and reached about 84% at 0.5 mM at 30 °C, also rising to 88% after 6 h of exposure. According to the polarization measurements, the investigated **APPH** works as a mixed-type inhibitor. Furthermore, the synergistic corrosion inhibition mechanism **APPH** showed that the inhibition efficiency maximizes with increasing inhibitor concentration, and the maximum value was 83% at 0.5 mM **APPH**. The adsorption of **APPH** on the LCS surface is more fitting to the Langmuir isotherm model. The free energy value (–Δ*G*° ads) was 33.3 kJ mol^−1^. Quantum chemical calculation was applied to **APPH** and acted as excellent support for the experimental data.

## Introduction

Metal corrosion has caused severe ecological impacts and economic loss to various productions, such as chemical engineering, oilfield category, desalination plants, etc. The destructive influence of strong acids such as Sulfuric acid (H_2_SO_4_) in the pickling and descaling process of metals seriously affected some infrastructure construction, especially carbon steel (CS) channels^1–3^ and accordingly, the addition of inhibitors is an important requirement^4,5^. Corrosion inhibition of steel is expected because of the excellent probability of protective film formation through the different electronic systems; π-bonds systems, free lone pairs on functional groups and conjugated systems between heterocyclic rings and functional groups^6–8^.

Corrosion inhibitors create a protective barrier on metal via chemical or physical adsorption^9–12^. Charge sharing or transfer of free lone pairs of electrons from the conjugated site of the inhibitor molecule to the non-complete d orbital on the metal surface is the chemical process that produces the protective layer^13–15^. Electrostatic forces between a steel surface charged in one direction and an inhibitor charged in the other direction are responsible for the physical formation of the protective layer. Like this, unoccupied d-orbitals of iron can share electrons with p-orbitals in aromatic systems to form feedback bonds, creating many chemisorption active sites^16,17^.

Phthalazinones are an intriguing class of organic compounds with a 2*H*-pyridazin-3-one core that includes aromatic systems, nitrogen atoms, and various electronegative centers that may be adjacent or substituent groups on the pyridazine ring^18–23^. In the past few years, derivatives of 2*H*-phthalazin-1-one were also applied as corrosion inhibitors of aluminum, steel and copper in an acidic environment^24–29^. Recently we considered the inhibitory outcome of two derivatives of 4-aryl phthalazinone, which contain amino or hydrazide moiety^30^.

Because not all individual compounds positively affect corrosion inhibition, many studies have been conducted to minimize the corrosion percentage by depending on the phenomenon of synergism through adding halide anions. It has been demonstrated that in acid solutions, halide ions form intermediates on a corroding steel surface, which may inhibit or accelerate iron anodic dissolution by substituting some of the adsorbed OH ions in the anodic process. Authors have proposed various mechanisms to explain the synergistic effect of organic inhibitors and halide ions. These include halide ion accumulation, which attracts the inhibitor molecule to the metal, halide ion and organic ion exchange, co-adsorption of inhibitor and halide ions, and a combination of various scenarios. According to the literature, inhibitor cation adsorption would be maximized on the directed dipoles formed by the halide ions as they first adsorb on the metal surface^31,32^.

As an important section of Theoretical chemistry, Quantum chemical calculations have proven to be an excellent tool for interpreting corrosion inhibition mechanisms^33–36^. The development of hardware and software of quantum chemical procedures, such as functional density theory (DFT), have recently been required as a quick and strong tool to interpret and explain corrosion inhibition performances of inhibitors problems. This is due to the strong associations found between the corrosion inhibitions effectiveness of maximum compounds and numerous semi-empirical criteria. The importance of the adsorption of inhibitor molecules on substrates in the context of corrosion research has lately increased^37–45^.

Further, the goal of this study is to continue our previous study on 4-aryl phthalazinone derivatives, which contain amino or hdrazide moiety to synthesize novel inhibitor^24^. **APPH** that contains both amino and hydrazide groups was investigated for its corrosion percentage on LCS in 0.5 M H_2_SO_4_ using electrochemical impedance spectroscopy (EIS), weight loss (WL), and potentiodynamic polarization (PDP) analyses. Iodide ions' synergistic impact on **APPH**'s inhibitive performance was also discussed. Also measured, discussed, and interpreted were a number of thermodynamic parameters, kinetic parameters, and quantum chemical calculations of density functional theory (DFT) for LCS corrosion at varied concentrations of **APPH**.

## Experimental procedure

### APPH synthesis and equipment

Opti Melt equipment (a melting point automated system with digital image) was used to measure the melting points and are uncorrected. Thin layer chromatography (TLC) on silica gel plates 60-F254 (Merck, 0.25 thickness layer) was used to determine the products' purity and monitor the reactions. Infra-red spectra FTIR were recorded on Bruker Model Vertex 70 with Platinum ATR unit. ^1^H-NMR spectroscopy were carried on Bruker 400 MHz in duterated dimethylsulphoxide (DMSO-d_6_) using tetramethylsilane (TMS) as an internal standard. Triple Quad LC/MS Agilent Technologies 6460 equipment with electrospray ionization (ESI-MS/MS) coupled to an Agilent Technologies 1260 using Agilent ZORBAX column (Eclipse plus C18; 4.6 × 100 nm × 3.5 µm), Mobile phase: 50% CH_3_CN/50% H_2_O + 0.1% HCOOH was used to detect Mass spectra. The **APPH** was synthesized according to the synthetic route in Scheme 1.

**Scheme 1:**
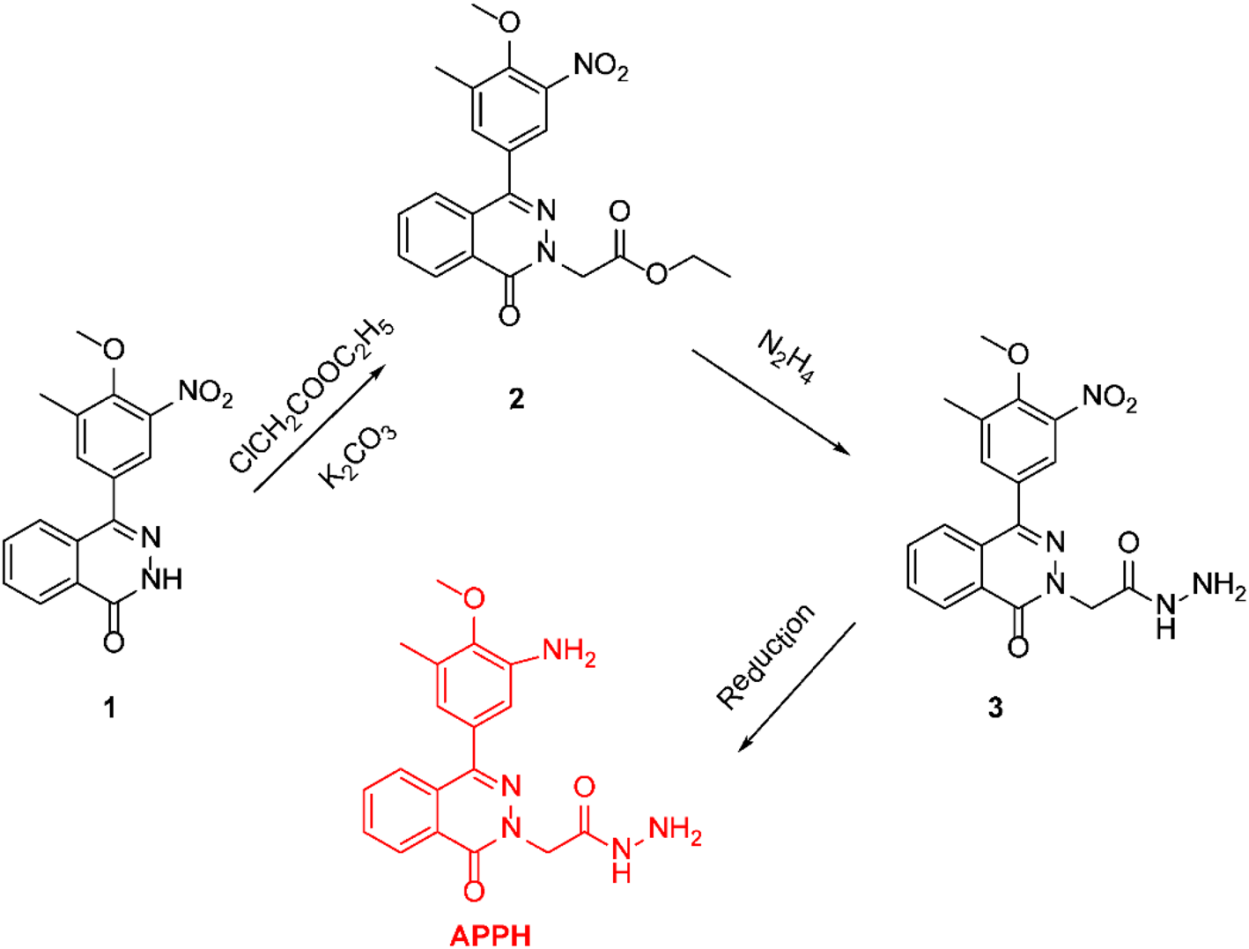
Synthetic root of **APPH** inhibitor.

**4-(4-5-mehyl-3-nitro) phenyl-2*H*-phthalazin-1-one** (**1**) was synthesized following the reported method^46^.

#### Ethyl [4-(4-mehoxy-5-methyl-3-nitro-phenyl)-1-oxo-1*H*-phthalaz-2-yl] acetate (2)

Compound **1 (**3.1 g, 0.01 mol) was reacted with ethyl acetoacetate (2.4 g, 0.002 mol) in acetone in the presence of anhydrous K_2_CO_3_ (2.6 g, 0.02 mol) at heating at reflux for 12 h (checked by TLC). The reaction mixture was put onto the water after the solvent was concentrated. Compound **2** was obtained with a 90% yield after the precipitate was filtered out, dried, and recrystallized from ethanol. m.p. 237–240 °C, FTIR (ν cm^−1^): 3316 (NH), 1672 (C=O, acetic acid hydrazide), 1650 (C=O, phthalazinyl), 1524, 1339 (NO_2_). ^1^H-NMR (400 MHz, DMSO-d6, ppm) δ: 2.44 (s, 3H, CH_3_), 3.92 (s, 3H, OCH_3_), 4.36 (bs, 2H, NH_2_), 4.78 (s, 2H, NCH_2_C=O), hydrazide_)_, 7.75–8.38 (m, 6H, Ar–H), 9.29 (bs, 1H, NH–NH_2_). MS *m*/*z*: 384 (M^+^ + 1).

#### [4-(4-Mehoxy-5-methyl-3-nitro-phenyl)-1-oxo-1***H***-phthalaz-2-yl]acetic acid hydrazide (3)

Compound **2** (4 g, 0.01 mol) was reacted with 2 mL hydrazine hydrate in 50 mL absolute ethanol at reflux for 4 h followed by cooling to room temperature. The obtained solid was filtered off followed by crystallization from ethanol to produce compound **3** in yield 90% as off-white crystals. m.p. 237–240 °C. FTIR (ν, cm^−1^): 3316 (NH_2_), 1672 (C=O, acetic acid hydrazide), 1650 (C=O, phthalazinyl), 1524, 1339 (NO_2_). ^1^H-NMR (400 MHz, DMSO-d6, δ ppm): 2.44 (s, 3H, CH_3_), 3.92 (s, 3H, OCH_3_), 4.78 (s, 2H, NCH_2_C=O), 5.14 (s, 2H, NH_2_ hydrazide_)_, 7.75–8.38 (m, 6H, ArH), 9.29 (bs, 1H, NH–NH_2_).) MS *m*/*z*: 384(M^+^+1).

#### [4-(3-Amino-4-mehoxy-5-methyl phenyl)-1-oxo-1***H***-phthalaz-2-yl] acetic acid hydrazide (APPH)

0.01 mol of compound **3**, 30 mg FeCl_3_ and 3.0 g active charcoal were mixed in 50 mL 1,4-dioxan in 3 neck 250 mL round flask fitted with condenser ending with a balloon on the top. The steering mixture received 16 mL of hydrazine hydrate, which was added in drops. After four hours of addition, the reaction had reached a temperature of 60 °C. The system needs to be periodically opened as the reaction takes place to allow the surplus gases to escape. The reaction mixture was filtered out after it had finished (confirmed by TLC), and the resulting solution was evaporated. The target product, APPH, was obtained from recrystallizing the acquired precipitate from ethanol in an 80–90% yield with off-white crystals. m.p. 260–265 °C. FTIR ATR (ν cm^-1^): 3377 and 3177 (NH_2_), 1671 (C=O). ^1^H-NMR (400 MHz, DMSO-d6, ppm) δ: 2.24 (s, 3H, CH_3_ Ar), 3.69 (s, 3H, OCH_3_), 4.89 (s, 2H, N–CH_2_–C=O, 5.08 (bs, 2H, NH_2_–NH), 6.76 (bs, 2H, ArNH_2_), 7.76–8.37 (m, 6H, Ar–H), 10.36 (bs, 1H, CH_2_–C=ONHNH_2_). MS *m*/*z*: 354 (M^+^+1).

### Corrosion study

#### Apparatus, materials and solution preparation for corrosion measurements

The electrochemical studies (EIS and PDP) were examined at room temperature via a Gamry Potentiostat/Galvanostat (Model reference 3000). The electrochemical cell was a conventional three-electrode system used a saturated calomel electrode (SCE) as a reference electrode, graphite rod as a counter electrode and steel rod as the working electrode in wt % was as follows; 98.62 Fe, 0.68 C, 0.662 Mn, 0.015 P, 0249 Si, 0.022 S, 0.027 Ni, and 0.031 Cu. The steel was mechanically cut in standard cylindrical coupons with a total surface area of 4.08 cm^2^ and used for weight loss measurements; Teflon covered other cylindrical coupons, and only one exposed surface with an exposed area of 0.64 cm^2^ for the electrochemical study. Then, samples were polished using SiC paper of various grades (#220 to #1200), washed with double-distilled water, degreased with acetone in an ultrasonic bath for 5 minutes, and air-dried before use. The pure H_2_SO_4_ of 98 percent analytical quality was used to create the corrosive medium containing 0.5 mol L^−1^ of H_2_SO_4_.

#### Technical conditions for corrosion measurements

##### Electrochemical measurements conditions

EIS experiments were performed at measured EOCP using a sinusoidal voltage signal of 10 mV peak to peak. The analysis was carried out in the frequency range of 0.1 Hz to 100 kHz. The PDP test was performed using a potential range of − 250 to + 250 mV versus SCE at EOCP with a sweep rate of 0.1 mV s^−1^. Each test was replicated at least three times to ensure reproducible results. The experimental data were analyzed using Echem Analyst 6.0 software.

##### Gravimetric study conditions

The calculated average values of low carbon steel (LCS) coupons were achieved by immersion of steel samples into 0.5 M H_2_SO_4_ using calculated concentrations of **APPH** for different periods at 303 ± 2 K. LCS coupons were removed after a specific amount of time, rinsed with distilled water and acetone, and then dried in a low oven before being reweighed. Using mathematical relationships, the corrosion rate, *C*_R_ (mg cm^−2^ h^−1^), and inhibition efficiency (*IE*) were determined from the weight loss of the examined LCS coupons using equations (1, 2):

1 $${C}_{R}=\frac{\mathrm{\Delta m}}{\mathrm{At}}\times 365$$

2 $$\mathrm{E\%}=\left(\frac{{C}_{{R}_{0}}-{C}_{R}}{{C}_{{R}_{0}}}\right)\times 100$$

where *C*_R0_ and *C*_R_ are the corrosion rates of LCS due to the dissolution in 0.5M H_2_SO_4_ mixed with calculated concentrations of **APPH**, respectively.

### Surface study

LCS samples were exposed to 0.5 M H_2_SO_4_ containing calculated concentrations of **APPH**. After removing the samples from the corrosive solution and drying it, the surface morphology and structure were investigated using a scan electron microscope (SEM lined to energy dispersive X-ray (EDX) spectroscopy (JEOL-JSM-5300LV, Tokyo, Japan) and Brucker FTIR. The same techniques should be used in the case of a synergistic effect.

### Adsorption isotherm and determination of adsorption thermodynamics parameters

The mechanism by which organic inhibitors attach to the LCS surface is discussed in the adsorption isotherm. Fitting the linear isotherm models (Frendlich, Langmuir, Frumkim, Tempkin, and Flory Huggins isotherm models) expressed in linear equations with the corrosion rate (*C*_R_) and the percentage of *Ɵ* of the **APPH** using the following Eqs. (3–9)^47–51^.

The Langmuir adsorption isotherm model (Eq. 3):

3 $$\frac{{C}_{R}}{\theta}=\left(\frac{1}{{K}_{ads}}\right)+{C}_{R}$$

Frumkim adsorption isotherm model (Eq. 4):

4 $$\mathrm{log}[{C}_{R}\left(\frac{\theta}{1-\theta}\right)=2\propto \theta +2.303log\,\,{K}_{ads}$$

Temkin adsorption isotherm model (Eq. 5):

5 $$\theta =\mathrm{ln}\,\,{C}_{R}+{K}_{ads}$$

Freundlish adsorption isotherm (Eq. 6):

6 $$log\theta =log\,\,{K}_{ads}+\mathrm{nlog}\,\,{C}_{R}$$

Flory–Huggins adsorption isotherm (Eq. 7):

7 $$\mathrm{log}\,\,(\frac{\theta }{{C}_{R}})=b\,\,\mathrm{log}\left(1-\theta \right)+\mathrm{log}\,\,{K}_{ads}$$

8 $${K}_{ads}={K}^\frac{1}{y}$$

El-Awady’s thermodynamic/kinetic adsorption isotherm model (Eq. 9):

9 $$log\frac{\theta }{1-\theta }=y\,\,log{C}_{R}+log\,\,K$$

Change of adsorption Gibb’s free energy (*ΔG*_ads_) is expressed in Equation (10) and was used to clarify the ability and nature of the adsorption. *K*_ads_ is a constant of the adsorption equilibrium that was achieved from the isotherm models.

10 $$\Delta {G}^{o}=-RTLnK$$

## Results and discussions

### Weight loss measurements

The effects of **APPH** concentration on steel LCS corrosion are shown in Table 1. According to the findings, adding **APPH** to 0.5 M H_2_SO_4_ dramatically reduces the corrosion rate (*C*_R_) of LCS while increasing the *IE*%. In the presence of APPH, a maximum IE of 92% was obtained at 0.5mM. Adding a higher concentration of APPH had no discernible effect on inhibition efficiencies above the used concentrations. As a result, 0.5 mM is chosen as the optimum concentration and used in subsequent immersion time studies. The enhanced performance of **APPH** at very low concentrations may be attributable to the hydrazide group's interaction with the steel surface through N or O atom and NH_2_.

**Table 1:** Effect of APPH concentrations on the corrosion rate of carbon steel in 0.5 M H_2_SO_4_.

[APPH] (mM)	Weight loss (g) *1 day*	Weight loss (g) *3 days*	Surface area (cm^2^)	*CR *(g/cm^2^.year) *1 day*	*CR *(g/cm^2^.year) *3 days*	*E (%)* *1 day*	*E (%)* *3 days*
0.0	0.3746	1.4686	4.08	5.69704	22.33496	0	0
0.05	0.2461	0.5851	4.08	3.7427	8.89916	34	60
0.1	0.1365	0.2849	4.08	2.07594	4.33285	63	81
0.2	0.0765	0.2549	4.08	1.16344	3.8766	79	83
0.4	0.0718	0.1779	4.08	1.09196	2.70556	81	88
0.5	0.0542	0.1124	4.08	0.82429	1.70942	86	92

### Electrochemical study

#### Tafel extrapolation technique

**Figure 1:**
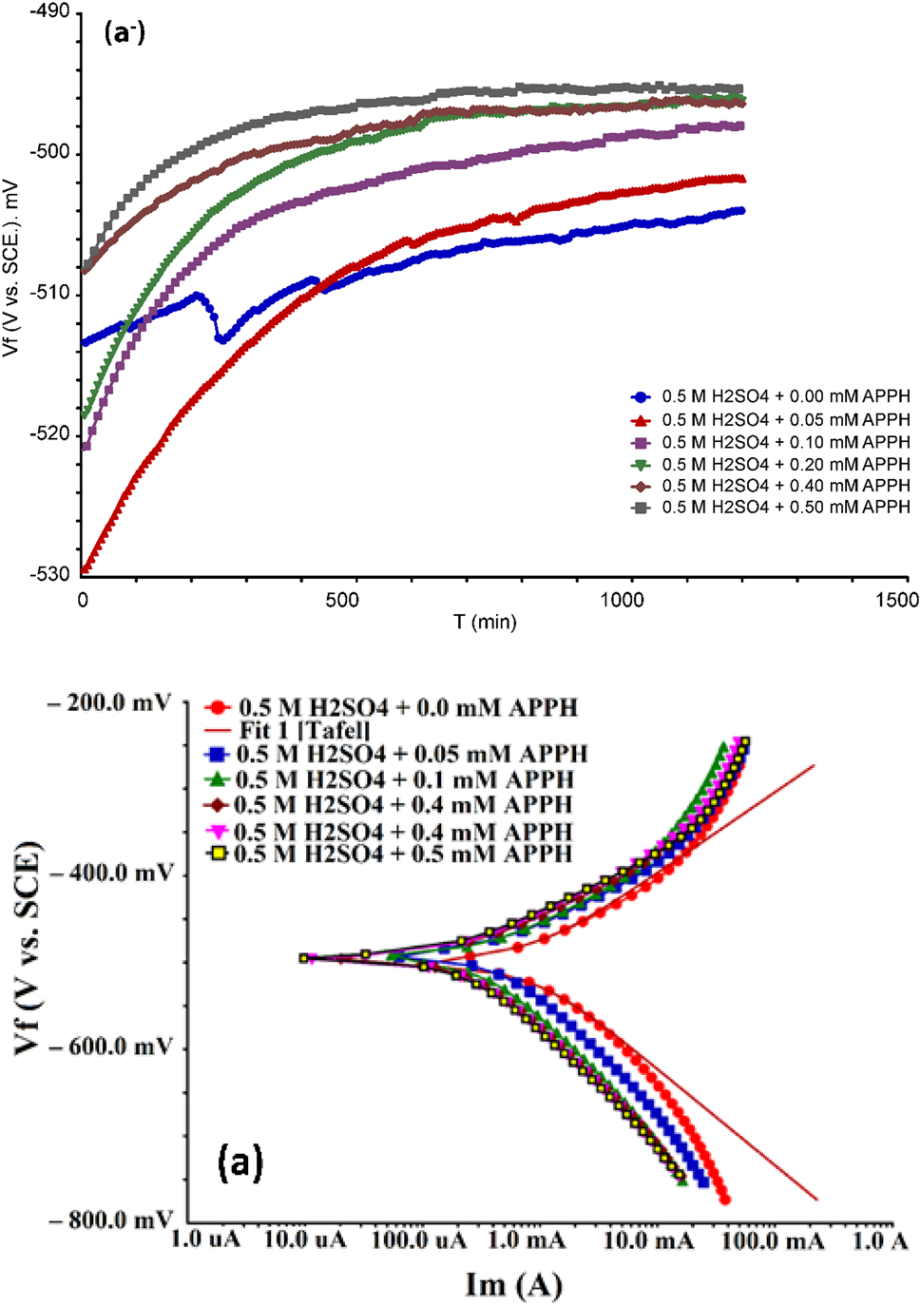
(**a**^−^) OCP and (**a**) Tafel polarization grave for LCS in 0.5 M H_2_SO_4_ with different concentrations of **APPH** at 303 K.

Figure 1 displays the polarization graphs of LCS in 0.5 M H_2_SO_4_ with different **APPH** concentrations. The electrochemical properties are summarized in Table 1. The findings indicated that the **APPH** molecule is an effective corrosion inhibitor since it demonstrated a steady decrease in corrosion current density relative to **APPH** concentration while increasing inhibition efficiency. The fact that all of the displacements are less than 85 mV and that *E*_corr_ is minimally shifted shows that the **APPH** molecule functions as a mixed-type inhibitor^52,53^. Table 2 shows that the inhibitory efficiency of **APPH** varies from 45 to 79% as concentration increases, with 0.5 mM being the ideal concentration. Further evidence that the **APPH** compound works as a corrosion prevention agent by lowering the polarization potential came from the reduction in Tafel slopes data from the cathodic and anodic areas. While reducing hydrogen evolution at the cathodic site, the Tafel slopes, on the other hand, have validated metal oxidation at the anodic site^54–56^. Additionally, because the values of *i*_corr_ and *C*_R_ decreased, the Tafel slopes without inhibitor values were larger than those cited in the absence of **APPH**.

11 $${C}_{R}\left(mpy\right)=t\times M\times \frac{{i}_{corr}}{F}$$

where M is the equivalent molar weight of iron, *i*_corr_ is the corrosion current density (A cm^−2^), *t* is the immersion time (*s*), and *F* is the Faraday constant^57^.

**Table 2:** Tafel polarization parameters were obtained at different concentrations of **APPH** for LCS in 0.5 M H_2_SO_4_ at 303 K.

[APPH] (mM)	–*E*_corr_	*I*_corr_ (µA)	*β*a	*β*c	*E* %
0.00	500	900	57.1	99.80	0
0.05	491	480	82	140	45
0.10	495	364	78	153	59
0.20	493	158	68	131	72
0.40	491	222	68	120	75
0.50	492	189	72	119	79

#### Measurements made using EIS

**Figure 2:**
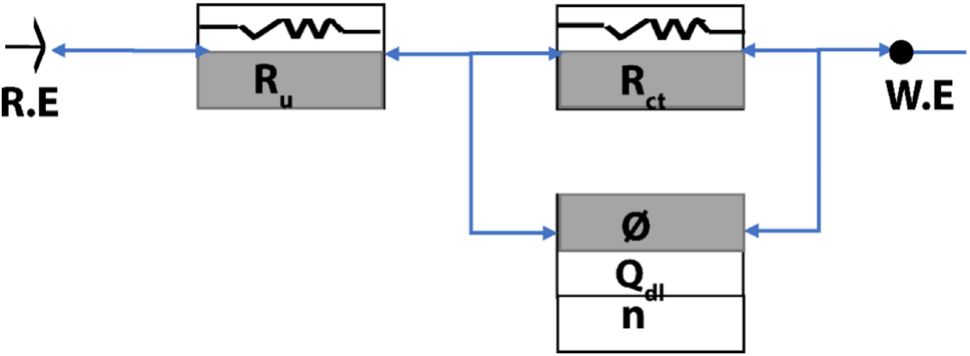
An inhibited system's equivalent electrical circuit (CPE) with an inhibited system.

*EIS* is a supplemental method for testing the affectivity of **APPH**, which is used to cover the surface of LCS in 0.5 M H_2_SO_4_ and to clarify the surface chemistry and kinetic properties of the LCS/electrolyte interface processes. Diverse corrosion systems, such as charge transfer regulation, diffusion control, or a mixed type, may exhibit different characteristics in their EIS analysis. EIS data is typically converted into equivalent electrical circuits in practice, which are then used to categorize the electrical properties of the electrochemical boundary. One of these circuits is the constant phase element model (CPE)^58^, which is broken down into three components CPE, solution resistance (*Rs*), and charge transfer resistance (*R*_ct_) (Fig. 2).

**Figure 3:**
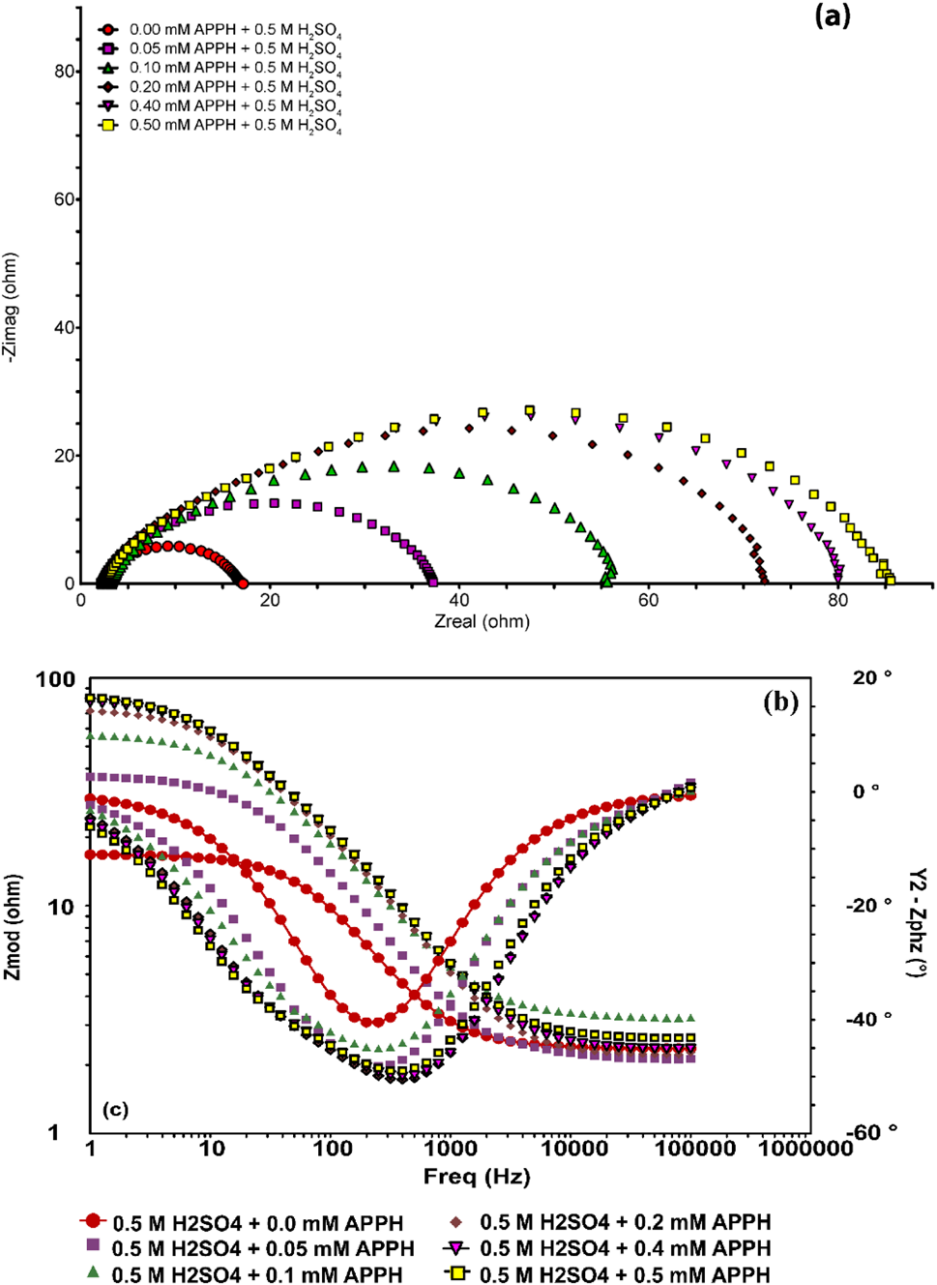
(**a**) Nyquist grave and (**b**) bode plots grave of LCS obtained at different **APPH** concentrations in 0.5 M H_2_SO_4_ at 303 K.

No nature effect of impedance diagrams with the presence of **APPH** with and without 100 mM KI compared with 0.5 M H_2_SO_4_; accordingly, the existence of **APPH** does not affect the corrosion mechanism (Table 3). The Nyquist grave and bode plot lines grave for LCS in 0.5 M H_2_SO_4_ electrolyte at calculated quantities of **APPH** are depicted in Fig. 3a,b. The observed single depressed capacity semicircles on the obtained plots for the LCS/electrolyte interface in the analyzed sulfuric acid environments with and without varied **APPH** amounts suggest that a charge transfer mechanism structures the corrosion behavior on the surface of LCS. The protective layer and adsorption formation at the LCS-electrolyte interface are connected to how the size of the Nyquist semicircle changes as **APPH** concentration increases^59^.

**Table 3:** The current comparable circuit simulation settings and associated inhibition efficiency (IE) of LCS in 0.5 M H_2_SO_4_ containing different **APPH** concentrations at 303 K.

[APPH] (mM)	*R*_s_ (ohm)	*R*_ct_ (ohm)	*I*_cor_	n	Goodness of fit × 10^−6^	*IE%*
0.00	2.3	15	461	0.88	72.27	0
0.05	2.9	35	379	0.79	533	58
0.10	3.1	54	384	0.79	632	73
0.20	2.2	71	388	0.83	598	77
0.40	2.4	79	391	0.92	963	82
0.50	2.3	91	463	0.87	798	84
0.05 + 100 mM KI	6.0	107	355	0.80	221	86
0.10 + 100 mM KI	2.5	116	424	0.88	467	87
0.20 + 100 mM KI	4.8	143	519	0.71	313	90
0.40 + 100 mM KI	1.9	160	580	0.69	925	91
0.50 + 100 mM KI	2.4	194	772	0.77	1480	93
0.40 + 1 h	5.1	98	420	0.79	980	85
0.40 + 2 h	3.5	116	579	0.83	1020	87
0.40 + 6 h	4.8	123	613	0.91	1350	88

### Synergism consideration

**Figure 4:**
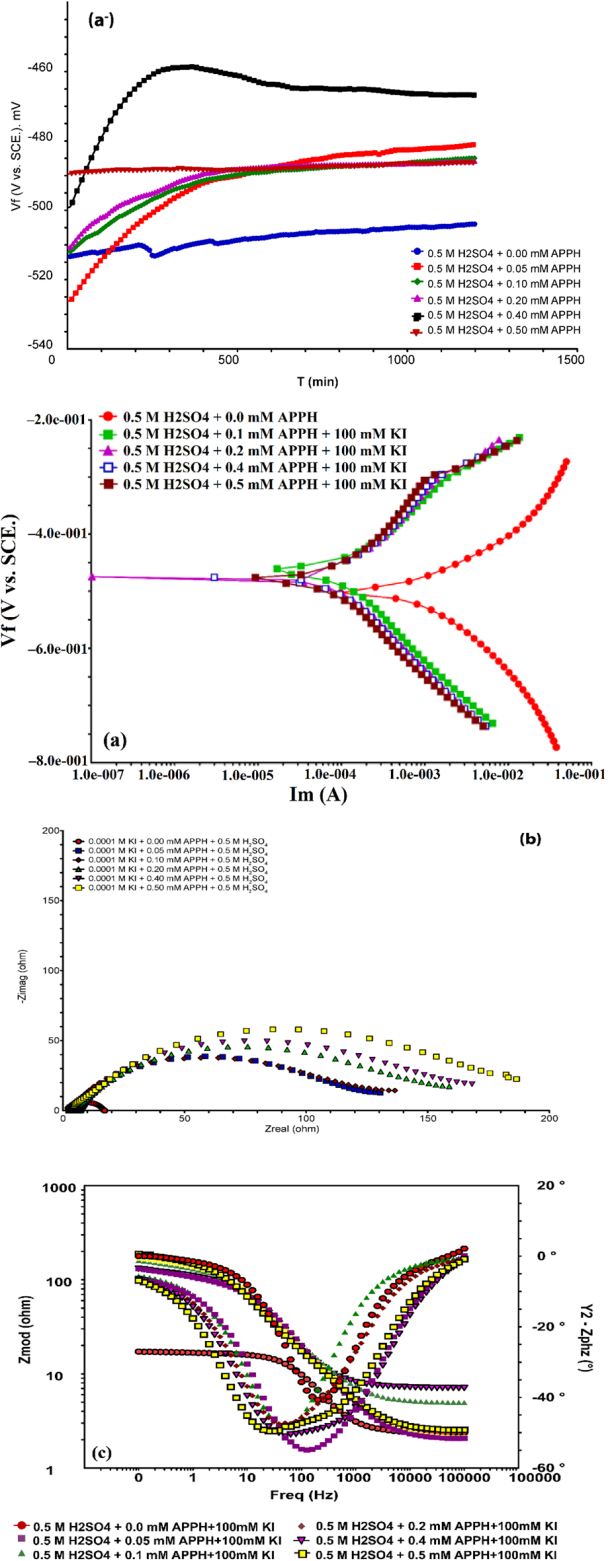
Synergistic effect of KI + **APPH** on LCS corrosion in 0.5 M H_2_SO_4_ using (**a**^−^) OCP (**a**) Tafel; (**b**) Nyquist grave; and (**c**) Bode plots grave.

When the combined effect of multiple compounds is greater than the sum of the activities of the individual compounds, this is known as the synergistic effect of **APPH** inhibitors. For the purpose of determining the synergism parameter (*S*), the formula that follows should be utilized as in equation (12).

12 $$S=\frac{1-(\eta 1+\eta 2)}{1-{\eta }_{1+2}^{^{\prime}}}$$

where *η*^1^ is the inhibitory action of iodide, *η*^2^ is the *IE* of the **APPH** and ${\eta }_{1+2}^{^{\prime}}$ is *IE* of iodide + **APPH**. The values of *S* are calculated as 1.81, 1.79, 1.83 and 1.81 with respect to 0.1, 0.2, 0.4 and 0.5 Mm **APPH**, respectively, which are more than unity, showing that the enhanced *IE* is also a function of KI^60^. Addition KI into 0.5 M H_2_SO_4_ corrosive media, I^−^ anion quickly absorbs into the anodic area of the LCS; thus, the positive excess charge on the anodic area of the steel surface is reduced, and the surface will be negatively charged. Accordingly, the protonated **APPH** is attracted to the negative surface of the steel, forming a protective layer through physical adsorption. The previous investigation approves the shift in corrosion potential to a less negative value; thus, the inhibition in the case of adding KI is an anodic inhibitor (Tables 2, 3; Fig. 4).

### ***The influence of exposure duration on the corrosion behavior of LCS in 0.5 M H***_***2***_***SO***_***4***_

**Figure 5:**
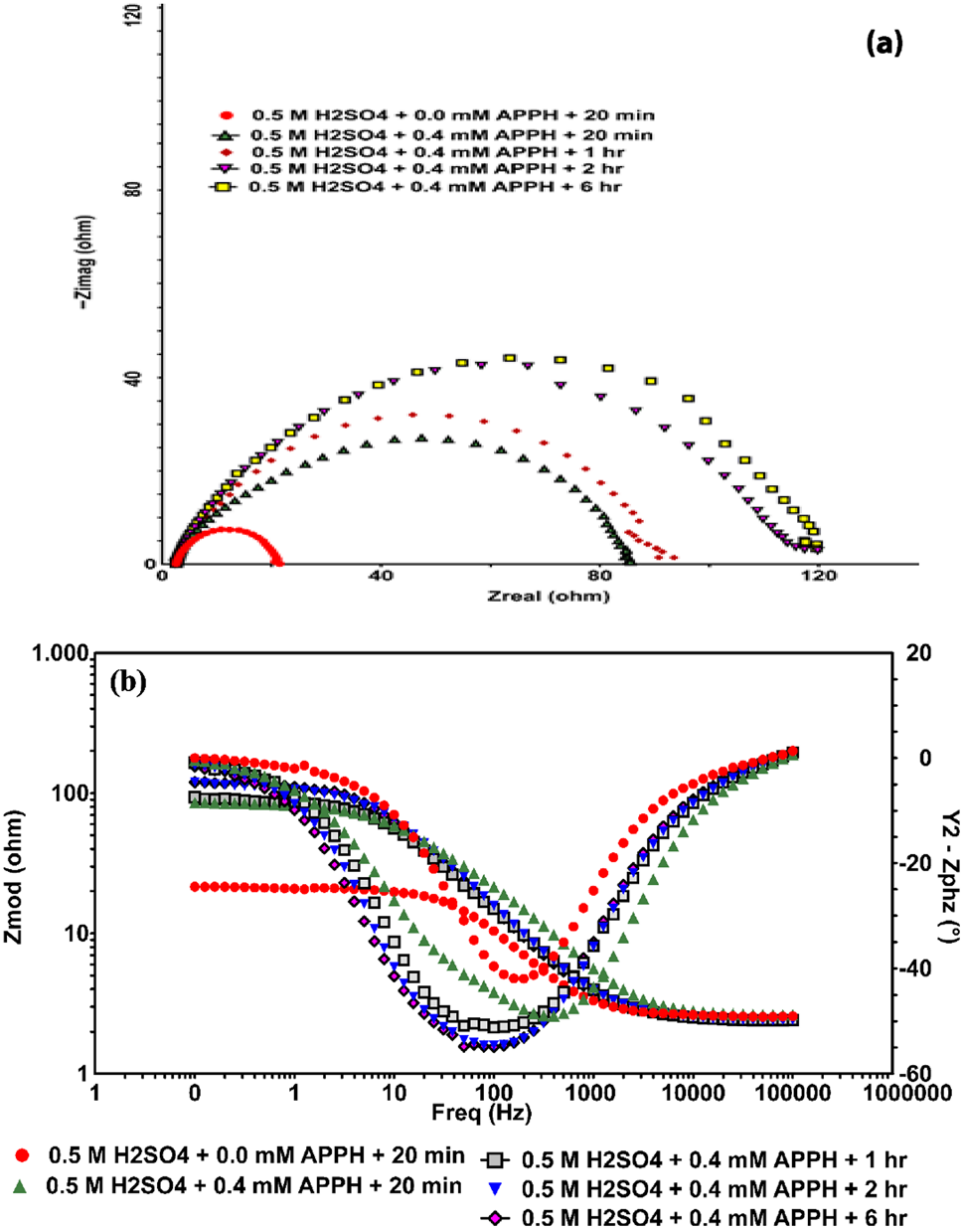
Impact of 6 h Immersion period of LCS corrosion in 0.5 M H_2_SO_4_ containing 0.4 mM **APPH** (**a**) grave of Nyquist; and (**b**) grave of bode plots.

In the absence of **APPH**, an increase in the immersion time to 1, 2, and 6 h led to an increase in the corrosion of the LCS, as shown by an increase in the values of *i*_corr_ and a decrease in the values of *R*_p_. This was confirmed by the fact that the values of *i*_corr_ increased while the *R*_p_ values decreased (Fig. 5). This is due to the fact that prolonging the duration of immersion results in a greater degree of LCS being dissolved by the caustic action of 0.5 M H_2_SO_4_ solution. According to some reports, the cathodic reaction for metals and alloys in H_2_SO_4_ solutions is the hydrogen evolution, which results in the consumption of electrons at the cathode. The increase in the anodic currents with potential and with the increase in immersion time indicates that increasing the applied voltage in a less negative direction makes it easier for steel to corrode^61^. The reduction in the corrosion parameters for the LCS was due to the inclusion of **APPH**. Whereas, the *i*_corr_ values go down while the *R*_p_ and *IE*% values go up when there is an increase in the amount of **APPH** present as well as when the exposure period of the LCS goes up from 0 to 6 h before the electrochemical measurements are taken. This was further corroborated by the electrochemical results shown in Table 3, which demonstrates that **APPH** is a good corrosion inhibitor for the LCS when immersed in a solution containing 0.5 M H_2_SO_4_ and its effectiveness rises with the increase of immersion time.

### Surface study

**Figure 6:**
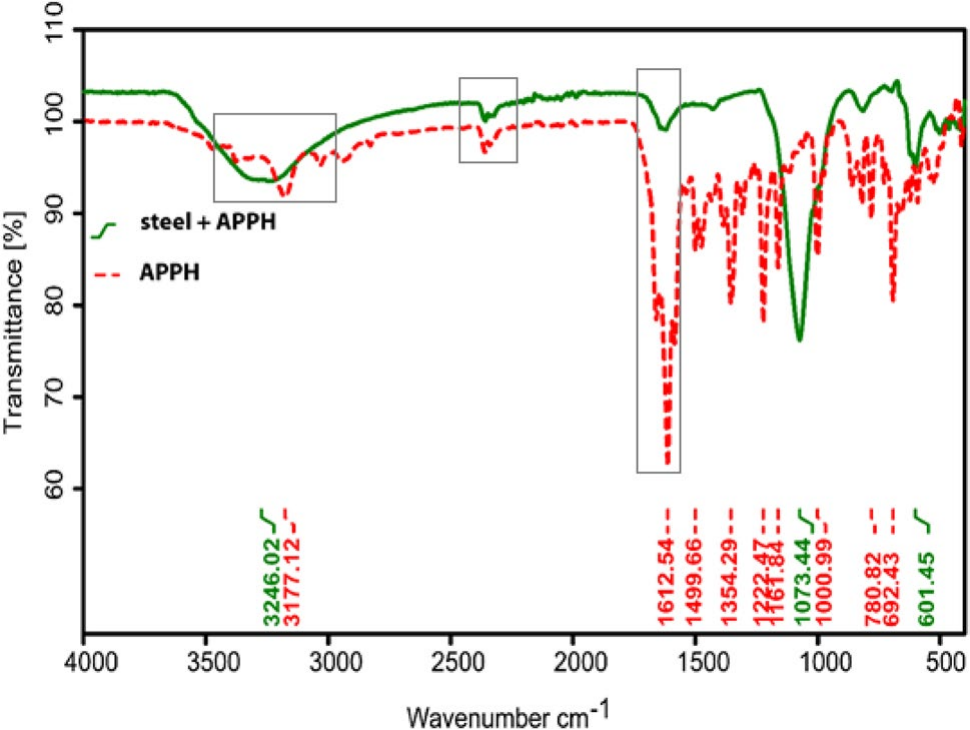
IR spectrum of **APPH** separately and on the LCS surface in 0.5 M H_2_SO_4_.

FTIR spectra were analyzed so that researchers could better understand the interaction of **APPH** molecules with the steel surface. Figure 6 displays the infrared (FTIR) spectra of pure **APPH** as well as scrapped samples collected from LCS surfaces following corrosion experiments conducted in the presence of **APPH**. It was discovered that the peaks that appear in the spectrum of pure **APPH** do not appear in the scrapped samples' spectra in the same way. The N–H stretching frequencies for **APPH** were observed to be at 3377 cm^−1^, the C=C stretching frequencies for individual **APPH** were recorded to be at 1612 cm^−1^, and almost completely disappeared with a noticeable reduction in peak integration in the scrapped sample. The stretching frequencies of the C–H, C–O group, almost disappeared in the scrapped sample.

**Figure 7:**
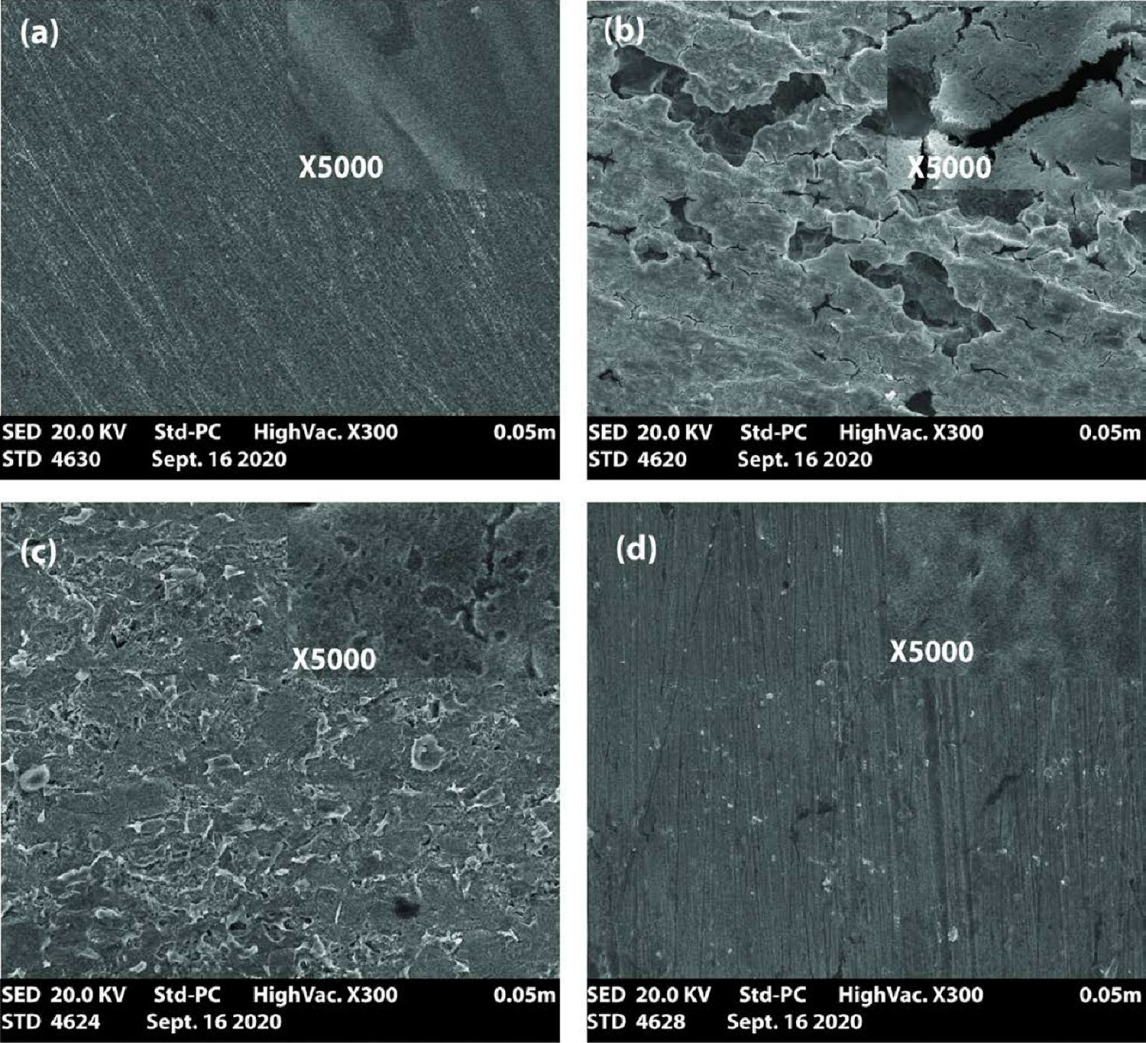
The SEM image of LCS specimens of (**a**) before treatment with 0.5 M H_2_SO_4_; (**b**) corroded; (**c**) inhibited by **APPH**; and (**d**) inhibited by **APPH** with 100 mM KI.

The surface morphology of the corroded coupon was characterized with the aid of SEM after immersion in 0.5 M H_2_SO_4_ solution and is a function of 0.5 mM **APPH** and in the case of adding 100 mM KI. The surface images presented in Fig. 7 are indicated that the surface of LCS before and after immersion in 0.5 M H_2_SO_4_ solution while LCS, which corroded in 0.5M H_2_SO_4_, exhibit a more severe grain border attack than the presence of 0.5M H_2_SO_4_ (Fig 7a,b). The attack is more extreme in the case of addition **APPH** (Fig. 7c) than in the addition of **APPH** and 100 mM KI (Fig. 7d). Metals and alloys often have the lowest energy and are most susceptible to corrosion attack near grain boundaries and areas of discontinuity. They offer **APPH** microcell molecules to adsorb at grain boundaries and discontinuities, lowering the rate of corrosion and the amount of hydrogen evolution.

### Computational calculations

Quantum chemical calculations have been employed extensively to provide a better understanding, on a molecular level, of the relationship that exists between the structure of inhibitors and the activities that they perform. The chemical reactivity of the APPH inhibitor can be anticipated by using this method, which involves conducting an analysis of the quantum chemical indices. According to the frontier orbital theory, the reaction that takes place between reactants typically takes place on the HOMO and LUMO, and the creation of a transition state is controlled by an interaction that takes place between the frontier orbitals of the reactants. As a consequence of this, analyzing the distribution of HOMO and LUMO was necessary to discover the inhibition mechanism. On the one hand, the unoccupied d orbitals of the Fe atom have the ability to accept electrons^42,44,62,63^. Equations (13–19) provide a summary of the computed quantum descriptors, which include *E*_HOMO_, *E*_LUMO_, *E*_HOMO_ − *E*_LUMO_ energy gap (*ΔE*), dipole moment (*μ*) and total energy (*TE*), electronegativity (*χ*), electron affinity (*A*), global hardness (*η*), softness (*σ*), ionization potential (*I*). The overall electrophilicity, denoted by the symbol (*ω*), as well as the fraction of electrons that are transported from the inhibitor to the iron surface, is denoted by the symbol (*ΔN*).

13 $$\Delta E=ELUMO-EHOMO=I-A$$

14 $$-ELOMO=I$$

15 $$A=-ELUMO$$

16 $$\eta =\frac{I-A}{2}=\frac{ELUMO-EHOMO}{2}=\frac{\Delta E}{2}$$

17 $$X=\frac{I+A}{2}$$

18 $$\updelta =I/\upeta $$

19 $$\Delta N=\frac{{X}_{Fe}-{X}_{inh}}{2\left({\upeta }_{Fe} + {\upeta }_{inh}\right)}$$

**Figure 8:**
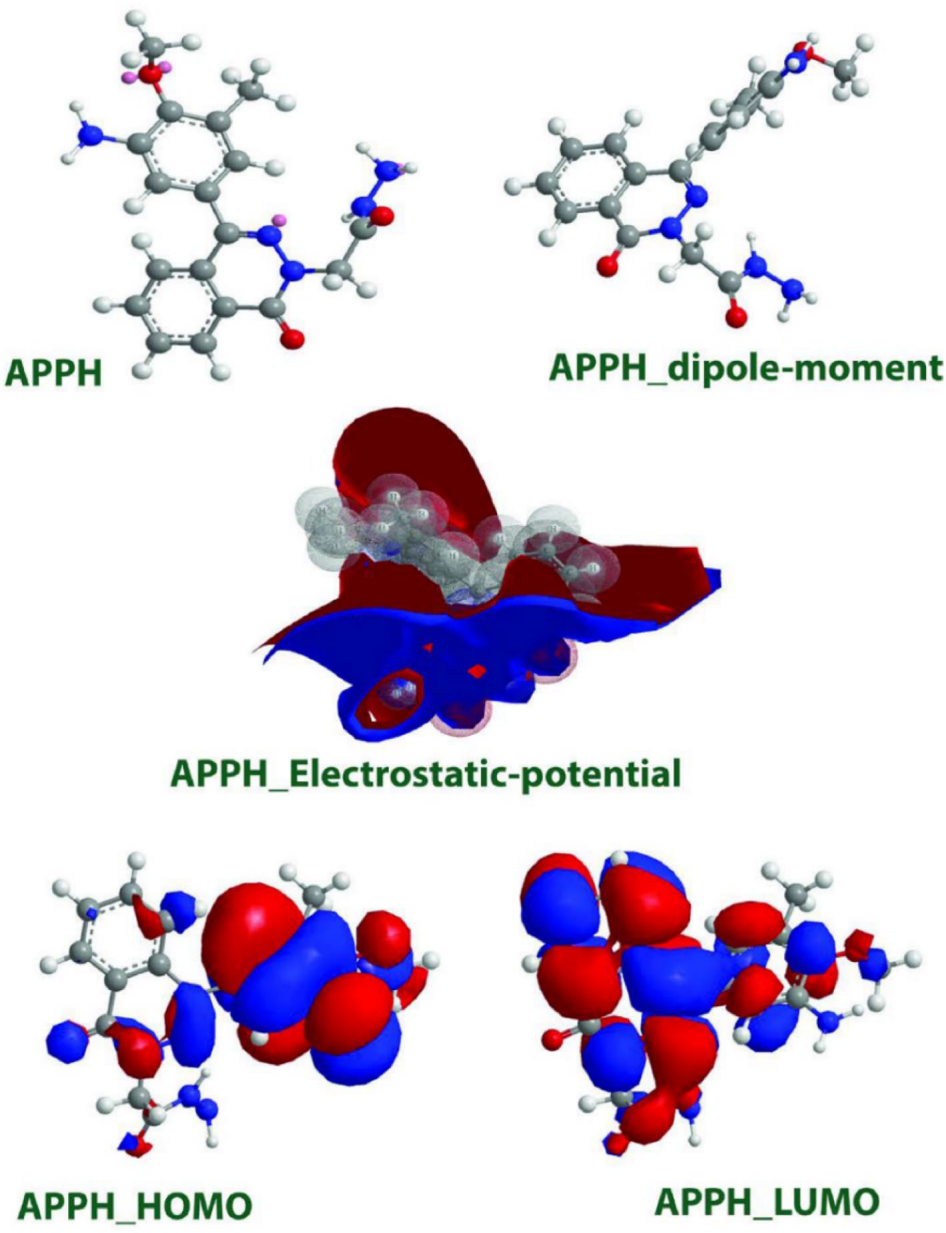
The frontier molecular orbital for protonated form of studied **APPH**.

Because **APPH** molecule contains hetero-atoms (N and O), a hydrazide group, and a benzene ring in addition to a benzene ring and is resonant on the whole **APPH** inhibitor molecule, the **APPH** corrosion inhibitor molecule has unique properties in terms of stability as well as the sensitivity of **APPH** molecule to the formation of coordination bonds with the LCS surface. These properties are due to the fact that **APPH** molecule is resonant on the whole inhibitor molecule. In the case of **APPH** molecule, HOMO and LUMO were investigated, and the results are presented in Fig. 8. Table 4 contains a listing of the *E*, *μ* and *χ*, and values that were calculated for the criterion energy of frontier molecular electrons. HOMO is the theory that describes how the contribution electrons of **APPH** molecule have an effect. It should come as no surprise that **APPH** molecule include more electrons. *E*_HOMO_ is a marker for inhibitive action and serves as a signal. Because **APPH** molecule also contains O and N in addition to the hydrazide group, the **APPH** particles used as a corrosion inhibitor have the ability to donate unshared pairs of electrons to the free orbitals of iron atoms, which are referred to as d-orbitals. A better explanation of LUMO could be found in the affinity calculations performed on **APPH** molecule. On the other hand, *E* is also an important quantity that specifies the bonding of **APPH** to the steel surface.

**Table 4:** The quantum chemical parameters of the APPH inhibitors that were examined.

Model	DFT31G	AM1	PM3
*E*_HOMO_ (eV)	− 0.2033	− 0.3105	− 0.3161
*E*_LUMO_ (eV)	− 0.0661	− 0.3267	− 0.0374
*∆E* (eV)	0.1372	− 0.0162	0.2786
*I* (eV)	0.2033	0.3105	0.3161
*A* (eV)	0.0661	0.3267	0.0374
*η*	0.0686	− 0.0081	0.1393
*δ*	14.5751	− 123.6094	7.1780
*μ* (Debye)	5.8158	−	−
*X*	0.1347	0.3186	0.1768
*∆N*	50.0316	− 412.9413	24.4886

A smaller value for E was associated with a more significant degree of inhibition in most cases. According to Table 4, **APPH** has an *E* value equal to 0.1372 eV. **APPH** has a minimal value of *χ*, demonstrating that it is quite effective at inhibiting activity. In this investigation, the value of *χ* supported the methodological findings. The high value of *μ* indicated that the corrosion inhibitor was superior to the others. According to the findings of this research, the value of the dipole moment *μ* of **APPH** molecules was 5.8158, which indicates that the **APPH** molecules have effective inhibitory control. As seen above, **APPH** molecules can adsorb on the LCS surface by exchanging water for more **APPH** molecule^5,64–67^.

The HOMO/LUMO for **APPH** showed that the HOMO orbital was localized on pyridine, but the LUMO orbital was switched to the benzene ring. DFT studies^65^ were used to estimate hardness and softness values. The **APPH** has a value of 0.0686, indicating that **APPH** as a corrosion inhibitor is expected to be a perfect inhibitor. **APPH** molecules have a chemical softness of 14.57, indicating that they have a higher inhibition efficiency^66^.

The calculations for HOMO and LUMO on **APPH** showed that the HOMO orbital was situated on the pyridine atom, whilst the LUMO orbital was moved to the benzene ring. This was discovered by comparing the two orbitals. To make estimations about the values of hardness and softness, DFT research was applied^65^. In light of the fact that the **APPH** has a value of 0.0686, one can conclude that the **APPH**, when employed as a corrosion inhibitor, ought to perform at the level of an ideal inhibitor. The chemical softness of **APPH** molecules is 14.57, which implies that they are more effective in suppressing activity than other molecules^66^.

### Adsorption isotherms

**Figure 9:**
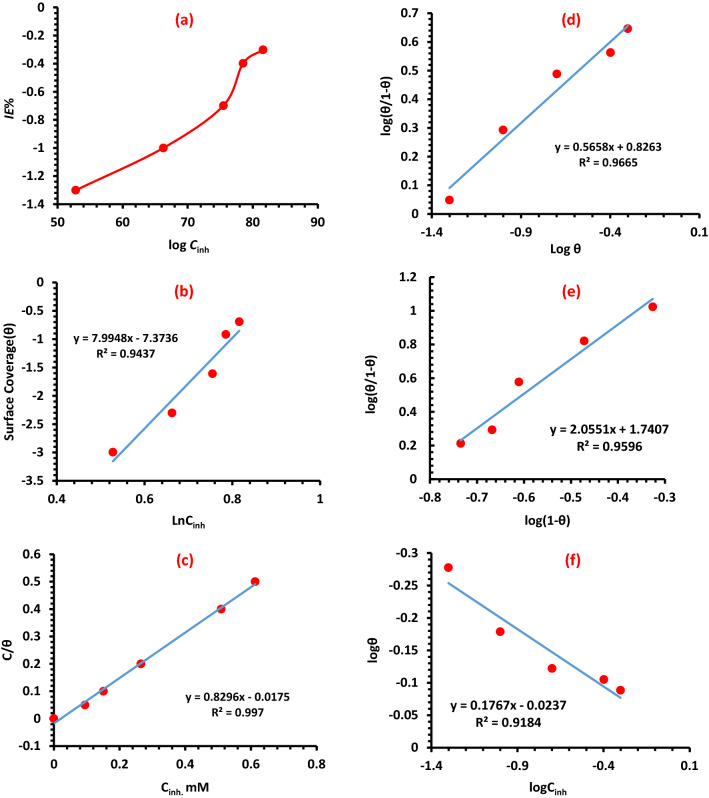
(**a**) Corrosion of carbon steel in 0.5 M H_2_SO_4_ solution and the relationship between the *IE*% and **APPH** concentration, (**b**) Temkin adsorption isotherm, (**c**) Langmuir adsorption isotherm plot, (**d**) Kinetic-thermodynamic model, (**e**) Flory–Huggins adsorption isotherm, and (**f**) Freundlich adsorption isotherm.

Figures 9 illustrates the different adsorption models that were investigated in this study. To choose the most suitable model, we considered the *R*^2^ values presented in Table 5 for each isotherm model. The data were compatible with the isotherms of Langmuir, Flory-Huggins, and Temkin, but the Langmuir isotherm offered the best fit for the data. The Langmuir isotherm, which has *R*^2^ values of 0.99, provides the most accurate description of the adsorption mechanism of **APPH** on LCS in a medium of sulfuric acid. As a consequence of this finding, the Langmuir adsorption isotherm is an appropriate tool for determining the adsorption equilibrium constant (*K*_ads_).

**Table 5:** *R*^2^ Values for the various adsorption isotherms considered for **APPH** at 303 K.

	Langmuir	Temkin	El− Awary’s	Flory–Huggins	Freundlich
*R*^2^	0.9970	0.9437	0.0.9665	0.0.9596	0.9184
K	57.14	7.994	4.826	112.202	1.4996

**Figure 10:**
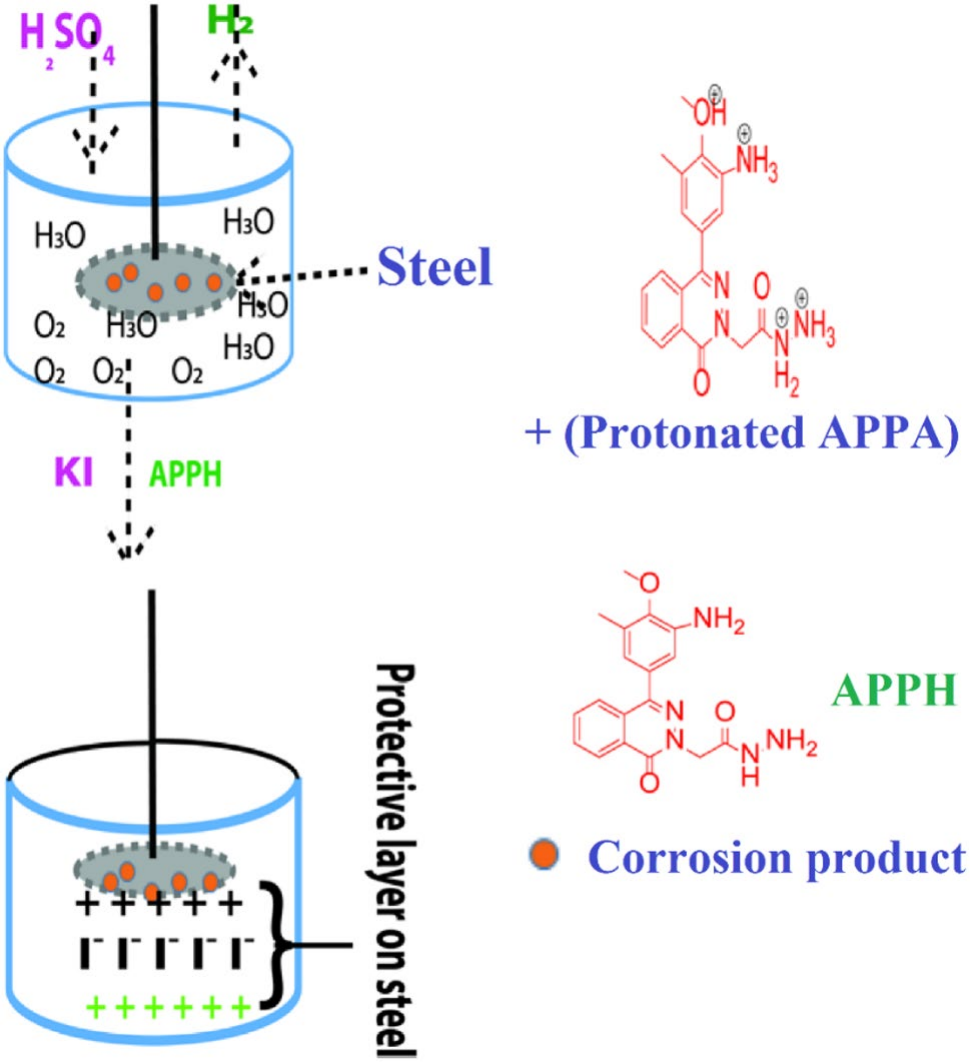
The mechanism of the **APPH** adsorption on the surface of LCS in 0.5 M H_2_SO_4_.

Table 6 reported the Gibb’s free energy change of adsorption (*ΔG*_ads_) at room temperature. The *ΔG*_ads_ of APPH as LCS corrosion inhibitor is negative and have value of 33.3 kJ/mol. This finding suggests that the APPH adsorption on the LCS surface occurred spontaneously, was possible, and instead followed the physical adsorption mechanism (Fig. 10).

**Table 6:** Thermodynamic parameters of (APPH) adsorption on LCS in 0.5 M H_2_SO_4_.

Kads (L/g)	*R*^2^	− ΔG°_ads_ (KJ mol^− 1^)
57.14	0.9970	33.63

When a metallic substrate is positively charged in H_2_SO_4_, many published works have shown that chloride ions and negative species first adsorb onto the surface of the substrate. Because of this, the negatively charged surface that was produced as a result is what makes the adsorption of protonated **APPH** possible via electrostatic attraction. It is important to point out that the protonation of the amino-functional group found in **APPH** results in a favorable state in H_2_SO_4_. Consequently, following the initial adsorption of I^1−^ and SO_4_^2−^ species, the protonated **APPH** will adsorb via electrostatic contact on the first created layer of the negative species. This will occur after the initial adsorption of I^1−^ and SO_4_^2−^ species. As mentioned earlier, **APPH** is considered a mixed adsorption inhibitor, which suggests that it inhibits both physisorption and chemisorption. Other un-protonated **APPH** induces coordination interactions with d empty molecular orbitals of metal, in addition to the apparent physical adsorption of the protonated molecule. Figure 10 shows a diagrammatic representation of the adsorption process in its basic form. Koumya et al.^67^ made observations that were very similar to these ones.

## Conclusion

[4-(3-Amino-4-mehoxy-5-methyl phenyl)-1-oxo-1H-phthalaz-2-yl] acetic acid hydrazide (**APPH**) was prepared and tested as a low carbon steel (LCS) corrosion inhibitor in 0.5 M H_2_SO_4_. The corrosion percentage of LCS in 0.5 M H_2_SO_4_ decreased with increases in the concentration of **APPH**, which is a mixed-type inhibition. **APPH** behaves as a mixed-type inhibitor and makes passivation. The adsorption of **APPH** has performed the inhibitory effect through the coordination bond of their heteroatoms with LCS surface. The adsorption of the **APPH** on the LCS follows the Langmuir adsorption isotherm model. The impact of exposure time using 0.4 mM of **APPH** shows that it maximized the inhibition efficiency to 88% after 6 hours of contact time. The value of free energy of adsorption (− ΔG° ads) was 33.3 kJ mol^−1^. The application of Quantum chemical calculations to **APPH** supported the experimental results.

## Data Availability

This article contains all of the data that was generated or processed while this study was being conducted, and it was published. It is recommended that anyone who is interested in requesting data from this study get in touch with Professor Dr. A. El Nemr.
